# Clinical Characteristics with an Impact on ADL Functions of PD Patients with Cognitive Impairment Indicative of Dementia

**DOI:** 10.1371/journal.pone.0082902

**Published:** 2013-12-09

**Authors:** Inga Liepelt-Scarfone, Monika Fruhmann Berger, Deborah Prakash, Ilona Csoti, Susanne Gräber, Walter Maetzler, Daniela Berg

**Affiliations:** 1 German Center of Neurodegenerative Diseases (DZNE), Tuebingen, Germany; 2 Department of Neurodegeneration, Hertie Institute for Clinical Brain Research, University of Tuebingen, Tuebingen, Germany; 3 Department of Neurology, Gertrudis Hospital, Leun-Biskirchen, Germany; Philadelphia VA Medical Center, United States of America

## Abstract

**Background:**

Dementia in Parkinson’s disease (PD) is defined as cognitive decline severe enough to affect activities of daily living function (ADL). The aim of our exploratory study was to compare two groups of PD patients. Both groups had cognitive deficits severe enough to justify diagnosis of dementia, but they differed according to caregivers’ rating on ADL dysfunction. Parameters which differed between the two groups were interpreted to affect the caregivers’ perception of ADL dysfunction in PD patients with cognitive impairment indicative of Parkinson’s disease dementia.

**Methodology/Principal Findings:**

Thirty of 131 Parkinson’s disease patients fulfilled the Movement Disorders Society Task Force – recommended, cognitive Level-I-criteria for dementia. According to standardized caregiver ratings, volunteers were grouped into 18 patients with (ADL-) and 12 without instrumental activities of daily living dysfunction (ADL+). Caregiver activities of daily living function ratings closely correlated with self-estimates of patients and those of physician (*p*<0.001). ADL- patients performed worse on tests assessing visual-construction (*p*<0.05) and attention (*p*=0.03) than ADL+ patients. Moreover, the postural instability and gait disorder subtype was more frequent in ADL- patients (*p*=0.009). ADL- patients tended to have more communication problems (*p*=0.05), more anxiety (*p*=0.05) and showed a tendency to be treated more often with neuroleptics (*p*=0.049) than ADL+.

**Conclusions/Significance:**

Results indicate that worse attention, visual-construction abilities, the postural instability and gait disorder subtype, communication problems, medication and presence of anxiety are related to activities of daily living dysfunctions in Parkinson’s disease patients with cognitive decline indicative of dementia. Our data suggests that not only cognitive factors but also non-cognitive factors seem to be linked to the diagnosis of Parkinson’s disease dementia associated with significant impact on instrumental activities of daily living function. Further studies with larger sample sizes are needed to verify our results.

## Introduction

Dementia is a frequent and disabling condition of idiopathic Parkinson’s disease (PD), affecting more than 80% of patients in advanced disease stages [1,2]. For diagnosis and treatment two clinical requirements are mandatory: significant cognitive impairment as defined e.g. by the Level-I criteria for probable Parkinson’s disease dementia (PDD) [3,4], and marked impact of cognitive dysfunction on Activities of Daily Living (ADL) functions. 

To date, minor cognitive dysfunction is the most common factor identified in various studies predicting PDD in the near future [2,5]. However, in early disease stages it is difficult to distinguish whether cognitive symptoms are a sign of later dementia or not. Indeed, cognitive worsening can also be found in PD patients who might not develop PDD [6]. A detailed ADL evaluation that detects relevant impairment is thus the most important part of the (differential) diagnosis of PDD. For an early detection of patients at risk for PDD it might therefore be helpful to identify, which clinical symptoms accompany cognitively driven worsening of ADL function. 

Information from caregivers about the patient’s behavior is often supposed to estimate the patient’s ADL function. However, evaluation of ADL dysfunction in PD is a challenging procedure as rater-specific aspects or non-cognitive factors can interfere with it [7–11]. Regarding the obvious impact of caregiver ratings on the ADL assessment of physicians, it is surprising that only few studies have addressed their accuracy so far [12–14]. Moreover, little is known about clinical signs and symptoms of PD patients, which most closely relate to caregiver ratings on cognitively driven ADL impairment. Previous results show that reduced attention is one of the strongest predictors for proxy reports on ADL dysfunction in patients already diagnosed with PDD [15]. 

Using a comprehensive test battery, we compared the clinical profile of 30 PD out of 131 patients who (i) had cognitive impairment indicative of probable PDD as identified by the Level-I screening procedure of the Movement Disorder Task Force (MDS-TF) [3,4] and (ii) were rated by their caregivers as having an ADL impairment (ADL-) or not (ADL+). The aim of this exploratory study was to identify clinical parameters which differentiate between the two PD groups, which might help the clinician to make the diagnosis of PDD. Both groups had cognitive deficits severe enough to justify diagnosis of PDD, but they differed according to the caregivers’ rating on ADL dysfunction. Parameters which differed between the two groups were interpreted to affect the caregivers’ perception of ADL dysfunction in PD patients with cognitive impairment indicative of PDD. 

## Materials and Methods

### Patients

We recruited a clinical sample of 131 patients with PD according to the United Kingdom Brain Bank (UKBB) Criteria [16]. Patients older than 50 years who reported that a caregiver would be willing to take part in the study were considered. Exclusion criteria were: diagnosis of dementia within the first year of parkinsonism, other central nervous system disorders, deep brain stimulation, history of drug or alcohol abuse, delirium and a Minimental State Examination (MMSE) [17] score<18 (cognitive testing not feasible, limited capacity to consent for study participation). Patients with signs of depression were not excluded as the influence of behavioral parameters was considered a relevant outcome parameter. All patients received their usual medication. The local ethical committee of the University of Tuebingen approved the study (121/2009BO2). All patients and caregivers gave written informed consent according to the Declaration of Helsinki.

### Classification of cognitive and ADL impairment

To identify patients with a substantial cognitive impairment indicative of probable PDD, we used the recommended screening procedure according to the cognitive Level-I diagnostic criteria of the MDS-TF [4].

First, we identified all patients who had a MMSE score<26 in addition to a cognitive impairment in at least two of four tasks defined as follows: (i) ≤3 of 5 points in the MMSE subtracting serial 7s task (attention), (ii) a verbal fluency performance (executive function) of z<-1.0 assessed by the German version of the CERAD (Consortium to Establish a Registry for Alzheimer's Disease, German version) [18], (iii) subscore = 0 in the drawing the pentagon task of the MMSE (visuo-construction); and (iv) ≤2 of 3 points in the 3-Word Recall of the MMSE (memory performance) (for details see 4). Therefore, patients’ grouping was based on the MMSE and the verbal fluency task, but not on further neuropsychological measurements or scales. Table 1 gives an overview on the comparison of patients who did not fulfill (n=101, PD non-demented, PDND) and those who fulfilled these cognitive Level-I criteria (n=30, Level-I cogn) indicative of PDD. 

**Table 1:** Neuropsychological performance of patients who did not fulfill (n=101, PD non-demented, PDND) and those who fulfilled the cognitive Level-I criteria (n=30, Level-I cogn.) indicative of PDD.

			**No cognitive impairment**		**Cognitive impairment indicating PDD**	
			**PD non demented** **(PDND)**		**Level-I-cogn.**	**PDND** **vs.** **Level-I-cogn.**
		**n**	**values**	**n**	**values**	p-corrected*
*Minimental State Examination*	screening	101	28 (23-30)	30	24 (15-25)	**<0.001**
*PANDA*	screening	100	21 (10-30)	27	10 (4-26)	**<0.001**
*Tower of London*	problem solving	101	38 (3-99)	30	7 (0-51)	**<0.001**
*Consortium to Establish a Registry for Alzheimer's Disease*						
Verbal fluency	word generation	101	34 (0-100)	30	11 (0-90)	**0.01**
Boston Naming Test	naming	101	50 (1-96)	30	6.5 (0-95)	**0.001**
Word-list memory	memory	101	34 (0-98)	30	3 (0-86)	**0.05**
Word-list recall	memory	101	31 (0-99)	30	12.5 (0-86)	**0.015**
Word-list recognition	memory	101	34 (0-86)	30	11 (0-92)	**0.005**
Word-list intrusion	memory	101	69 (0-79)	30	3 (0-84)	**0.005**
Praxis	visuo-construction	101	38 (0-95)	30	1.5 (0-86)	**<0.001**
Praxis-Delay	visuo-contruction/memory	101	21 (0-97)	30	1 (0-84)	**0.003**
Trail Making Test, Part A	psychomotor speed	101	62 (1-100)	30	1.5 (0-62)	**<0.001**
Trail Making Test, Part B	set shifting	101	73 (0-100)	30	0 (0-66)	**<0.001**
*Wechsler Memory Scale Revised*						
Logical Memory I	memory	101	22 (1-98)	30	3 (1-70)	**0.04**
Logical Memory II	memory	101	22 (0-92)	30	3.5 (0-68)	**0.08**
*Nuernberger-Alters-Inventory*						
Digit span	memory	101	65 (4-100)	30	27 (4-100)	**0.001**
Figure Test	visuo-spatial/memory	101	65 (4-95)	30	14 (0-88)	**0.001**
*Visual Object and Space Perception Battery*						
Object decision	visuo-spatial	101	38.1 (3.6-100)	30	11.9 (2.4-100)	**0.02**
*Berlin Apraxia Test*	praxis/executive function	101	39 (27-44)	30	30.5 (17-39)	**0.001**
*Test for Attentional Performance*						
Alertness-no cue, Median	attention/alertness	98	12 (0-79)	28	2 (0-50)	**0.001**
Alertness-with cue, Median	attention/alertness	98	14 (0-82)	28	2.5 (0-50)	**0.001**
Go-Nogo, Median	directed attention	101	54 (0-100)	29	4 (0-66)	**0.001**

Values are given as median and range (minimum-maximum). *P-values are corrected for age and motor performance according to the Unified Parkinson’s Disease Rating Scale Part III by using multivariate regression analysis. Mean group performances are given in relation to the standardized values provided by the test manuals with lower values indicating poorer test performances. Only for the Berlin Apraxia Test raw data are presented. ADL: Activities of Daily Living.; PANDA: Parkinson Neuropsychometric Dementia Assessment; PD: Parkinson's Disease; PDD: Parkinson's Disease Dementia; p: Level of significance; N: number.

 In a second step, the latter patients were subdivided according to their caregiver-rated ADL performance. ADL ratings were based on a widely used German proxy-scale (Nuernberger-Alters-Alltagsaktivitaeten-Skala, NAB) [19], assessing instrumental activity of daily living functions. Scores below z=-1.0 were considered as impaired ADL function (ADL-). 

### Neuropsychological, clinical, and behavioral assessment

The following parameters were assessed to compare the clinical characteristics of the two study groups:

#### Neuropsychological test battery

A comprehensive neuropsychological test battery comprised numerous standardized tests on all relevant cognitive functions in PD such as executive function, memory, visuo-construction and attention tasks (see Table 1). 

The Parkinson Neuropsychometric Dementia Assessment (PANDA) was used to screen for global cognitive impairment [20]. The Tower of London test (TL-D) [21] was included to assess planning ability. The German version of the CERAD [18] provides tasks on verbal fluency (VF for animals), naming ability (Boston Naming Test, BNT), word–list memory (WL), word-list recall after delay (WL-Delay), word-list recognition (WL-R) and on visuo-construction (Praxis) including a delayed recall (Praxis-Delay). The Trail Making Test (TMT) A and B, both part of the CERAD battery, were used to assess behavioral regulation, motor speed and set shifting [18]. 

Memory function was further examined by the Logical Memory I and II of the revised Wechsler Memory Scale [22]. Working memory capacity was evaluated by using the digit span part (DS), including both forward and backward recall, of the NAI (Nuernberger Alters-Inventory) [19]. The Figure Test (FT) [19] of the NAI was used to assess visual memory function. 

The object decision part of the Visual Object and Space Perception Battery (VOSP) [23] provides an assessment of visual function. The ideomotor part of the Berlin-Apraxia-Test (BAXT) [24] was used to quantify ideomotor apraxia. Attentional processes were recorded by two subtests of the computerized version of the TAP “Test for Attentional Performance” [25]. The subtest “alertness” consists of a single visual reaction time task (Alertness no cue) and an acoustic cued condition (Alertness with cue). The subtest “Go-Nogo” provides a test on selective attention. 

In both, the BAXT and the TAP, patients were instructed to take the dominant arm or the side with less parkinsonian signs. Analyses are based on standard norms (percentile rank scores) of healthy German control subjects as published in the manuals. Data are corrected either for age (NAI, WMS-R, VOSP) or for age and education (CERAD, TAP, TMT-A, TMT-B, TL-D; please compare [26]). 

### Neurological scales

Neurological evaluation included the Unified Parkinson’s Disease Rating Scale (UPDRS) part III [27], the Hoehn and Yahr (H&Y) scale [28] and the Schwab and England Activities of Daily Living Scale (S&E-ADL) [29]. Based on the UPDRS scores, patients’ were assigned to one of three different motor types: the postural instability and gait disorder (PIGD), the tremor-dominant, or the indeterminate subtype [30,31]. 

Levodopa equivalent dose (LEDD) was calculated using the following conversion factors: 100 mg levodopa equaled 125 mg levodopa sustained release, 1mg pergolide, 1mg pramipexol, 5 mg ropinirole, 5 mg rotigotin, 10 mg bromocriptine, 10 mg apomorphine, 1/5 entacapone, and 1.5 mg cabergoline. Additionally, 5% was added to the total levodopa dose for every 5 mg of selegiline or 1 mg of rasagiline, up to a maximum of 10%; [32–34]. 

#### Neurobehavioral assessment

We applied the Beck-Depression Inventory (BDI), which provides a self-rating scale for symptoms associated with depression. A value ≥18 indicates a major depression [35]. The additionally used Parkinson’s Disease Questionnaire (PDQ-39) [36] evaluates eight aspects of patients’ perceived health-related quality of life (HRQoL), i.e. mobility, ADL, emotions, stigma, social, cognitions, communication and body pain. Moreover, the Neuropsychiatric Inventory (NPI) [37] was used to evaluate ten neurobehavioral domains (e.g. delusions, hallucinations, depression or apathy). Corresponding to the NAB proxy scale, patients’ self-estimated ADL function was assessed by a standardized questionnaire (Nuernberger-Alters-Alltagsaktivitaeten-Skala, NAA) [19]. 

### Statistical Analyses

As data were not normally distributed, the data analysis was based on non-parametric statistics. For validation purposes (e.g. to prove clear differences in cognitive impairment), analyses between the PDND (n=101) and the total group of patients who met the Level-I-cogn criteria (n=30) were calculated. Linear regression analysis was used, with the parameters of interest included into the model as predictors and the group-membership (PDND vs. Level-I cogn criteria binary coded), age and UPDRS-III motor score included as independent variables. Disease duration was not included in the regression model, as it was highly correlated with the UPDRS-III score. 

Statistical mean group comparisons between the ADL- and ADL+ groups were performed with either the Mann-Whitney-U or the Fisher’s exact test. Due to the exploratory nature the significance level was set at *p*<0.05 (two-sided). All analyses were done with IBM SPSS Statistics 19.0 for Windows. 

## Results

Thirty of 131 PD patients (22.9%) fulfilled the Level-I criteria for the classification of cognitive impairment indicative of PDD. Of those, 12 were classified as ADL+ and 18 as ADL-. For validation purposes, the proxy reports on severity of ADL impairment were compared to ratings of physician and patients self-estimates. The NAB scale correlated well with evaluations of physicians (S&E-ADL *p*<0.001) and the patient’s NAA score (NAA *p*<0.001). 

### Missing Values

The PANDA was not performed in six patients (n=3 PDND, n=2 ADL+ and n=1 ADL-) due to reduced compliance and/or severity of cognitive impairment. Missing data in the two subtests of the TAP was due to technical problems (e.g. data saving errors). One patient in of the ADL- group refused to complete the BDI. 

### PDND versus Level-I-cogn total

Patients who fulfilled the Level-I-cogn criteria were significantly more impaired than the PDND group in nearly all neuropsychological tasks (all *p* values ≤0.05 corrected for age and motor performance, see Table 1 for details). These results strongly indicate that patients who met the cognitive MDS-TF screening criteria indeed suffer from a substantial cognitive impairment suggesting probable PDD. No significant differences (p>0.05) were found between the groups with regard to gender (60.4%), level of education (12 years, 8-19), and levodopa equivalence dose (504, 0-2030). The PDND group was younger (67.0 years, 51-85; p<.001), had a shorter disease duration (4.0 year, 0.3-19; p<.001) and lesser motor impairment according to the UPDRS-III (24.0 points, 7-62; p<.0.001) compared to the Level-I- cogn group. 

### ADL- vs. ADL+

Almost all patients (17 out of 18) who met the Level-I-cogn criteria and were ADL- belonged to the PIGD subtype (see Table 2). In comparison, only 50% of the ADL+ group were PIGD (*p*=0.009). Our neuropsychological assessment revealed that ADL- patients performed worse on neuropsychological tests assessing visuo-construction (*p*<0.05) and attention (*p*=0.03, Table 3). 

**Table 2:** Clinical data of patients with cognitive performance indicating PDD who were rated by rated by their caregivers as having an ADL impairment (ADL-) or not (ADL+).

		**No ADL impairment (ADL+)**		**ADLimpairment (ADL-)**	**ADL+vs.ADL-**
	**n**	**values**	**n**	**values**	**p**
**Demographics**					
Age at evaluation (years)	12	71.5 (63-85)	18	74 (64-86)	0.54
Male gender [N (%)]	12	9 (75.0)	18	14 (77.8)	1.00
Years of disease duration	12	7.5 (2-16)	18	10.5 (4-24)	0.09
Years of education	12	11 (8-20)	18	11 (8-17)	0.87
**Motor assessment**					
UPDRS-III	12	35.5 (19-57)	18	39.5 (28-60)	0.11
*Hoehn & Yahr stages [N (%)]*	12		18		
1 1.5		2 (16.7) 0 (0)		0 (0) 0 (0)	0.40
2 2.5		2 (16.7) 2 (16.7)		4 (22.2) 1 (5.6)	
3 4		3 (25.0) 3 (25.0)		7 (38.9) 6 (33.3)	
Schwab & England Scale	12	80 (50-100)	18	60 (10-80)	**0.005**
*Motor Type*	12		18		
Tremor-dominant [N (%)]		3 (25.0)		0	**0.009**
PIGD [N (%)]		6 (50.0)		17 (94.4)	
Indeterminate [N (%)]		3 (25.0)		1 (5.6)	
**Medication**					
Levodopa equivalence dose (mg)	12	450 (66.7-1360)	18	726.5 (100-1582.5)	0.22
Monotherapy with L-Dopa [N (%)]		4 (33.3)		5 (27.8)	**0.047**
Monotherapy with Dopamin-Agonists [N (%)]		3 (25.0)		0	
L-Dopa and Dopaagonists [N (%)]		5 (41.7)		13 (72.2)	
Other PD medication [N (%)]		0		0	
Antidepressants [N (%)]	12	1 (8.3)	18	5 (27.8)	0.36
Neuroleptics [N (%)]	12	1 (8.3)	18	8 (44.4)	**0.049**
Antidementiva [N (%)]	12	2 (16.7)	18	6 (33.3)	0.42
**Depression**					
Becks Depression Inventory	12	11.5 (0-34)	17	14 (1-39)	0.40
Major depression [N (%)]	12	2 (16.7)	17	4 (23.5)	1.00

Values are given as median and range (minimum-maximum) unless otherwise noted. ADL: Activities of Daily Living; N: number; mg: milligram; (%): percentage; p: Level of significance; PD: Parkinson's Disease; UPDRS-III: Unified Parkinson Disease Rating Scale Part III; PIGD: phenotype with postural instability and gait disorders; Indeterminate: phenotype with tremor as well as postural instability and gait disorders.

**Table 3:** Neuropsychological performance of patients with cognitive performance indicating PDD who were rated by rated by their caregivers as having an ADL impairment (ADL-) or not (ADL+).

			**No cognitive impairment**		**Cognitive impairment indicating PDD**		**Cognitive impairment indicating PDD**	
			**PD non demented** **(PDND)**		**No ADL impairment (ADL+)**		**ADL** **impairment (ADL-)**	**ADL+** **vs.** **ADL-**
		**n**	**values**	**n**	**values**	**n**	**values**	**p**
*Minimental State Examination*	screening	101	28 (23-30)	12	24 (19-25)	18	24 (15-25)	0.47
*PANDA*	screening	100	21 (10-30)	10	17.50 (9-26)	17	9.00 (4-18)	**0.002**
*Tower of London*	problem solving	101	38 (3-99)	12	10 (0-35)	18	3 (0-51)	0.09
*Consortium to Establish a Registry for Alzheimer's Disease*								
Verbal fluency	word generation	101	34 (0-100)	12	4.5 (0-76)	18	16 (0-90)	0.16
Boston Naming Test	naming	101	50 (1-96)	12	4 (0-76)	18	9 (0-95)	0.23
Word-list memory	memory	101	34 (0-98)	12	5.5 (0-42)	18	3 (0-86)	0.65
Word-list recall	memory	101	31 (0-99)	12	21 (0-86)	18	7.5 (0-69)	0.47
Word-list recognition	memory	101	34 (0-86)	12	3.5 (0-92)	18	12 (0-86)	0.29
Word-list intrusion	memory	101	69 (0-79)	12	3.0 (0-84)	18	3 (0-82)	0.83
Praxis	visuo-construction	101	38 (0-95)	12	2.5 (0-86)	18	0 (0-82)	**0.05**
Praxis-Delay	visuo-contruction/memory	101	21 (0-97)	12	15 (0-84)	18	0 (0-13)	**0.001**
Trail Making Test, Part A	psychomotor speed	101	62 (1-100)	12	1.5 (0-62)	18	1.5 (0-58)	0.52
Trail Making Test, Part B	set shifting	101	73 (0-100)	12	0 (0-58)	18	0 (0-66)	0.83
*Wechsler Memory Scale Revised*								
Logical Memory I	memory	101	22 (1-98)	12	3 (1-23)	18	3 (1-70)	0.63
Logical Memory II	memory	101	22 (0-92)	12	4 (0-47)	18	3.5 (0-68)	0.99
*Nuernberger-Alters-Inventory*								
Digit span	memory	101	65 (4-100)	12	27 (4-65)	18	31.5 (4-100)	0.48
Figure Test	visuo-spatial/memory	101	65 (4-95)	12	20.5 (0-82)	18	10 (0-88)	0.28
*Visual Object and Space Perception Battery*								
Object decision	visuo-spatial	101	38.1 (3.6-100)	12	34.6 (3.6-100)	18	9.5 (2.4-47.6)	0.08
*Berlin Apraxia Test*	praxis/executive function	101	39 (27-44)	12	29.5 (21-38)	18	32.5 (17-39)	0.33
*Test for Attentional Performance*								
Alertness-no cue, Median	attention/alertness	98	12 (0-79)	11	3 (0-46)	17	2 (0-50)	0.17
Alertness-with cue, Median	attention/alertness	98	14 (0-82)	11	4 (0-50)	17	2 (0-31)	0.75
Go-Nogo, Median	directed attention	101	54 (0-100)	11	10 (0-66)	18	1.5 (0-54)	0.03

Values are given as median and range (minimum-maximum). Mean group performances are given in relation to the standardized values provided by the test manuals with lower values indicating poorer test performances. Only for the Berlin Apraxia Test raw data are presented. ADL: Activities of Daily Living.; PANDA: Parkinson Neuropsychometric Dementia Assessment; PD: Parkinson's Disease; PDD: Parkinson's Disease Dementia; p: Level of significance; N: number.

There was a tendency for ADL- patients to be rated as more anxious by the caregivers (NPI-subscale-E: ADL-, median=0, range=0-8; ADL+, median=0, range=0-1; *p*=0.05) than ADL+ patients. They reported themselves as being slightly more impaired in ADL function (PDQ-scale 2: 62.5, 4.2-91.7, ADL+, 29.2, 0-100; *p*=0.03) and tended to have more severe communication problems (PDQ-39-communication: ADL-, 41.7, 0-66.7; ADL+, 25, 0-75; *p*=0.05) than the ADL+ group. ADL- patients received neuroleptics more often compared to patients with ADL+ (*p*=0.049), however this effect was statistically borderline. 

Even if the frequency of patients with major depression did not differ between the two groups (p=1.00), severity of depression might have affected the interpretation of the neuropsychological data. Therefore, analysis of the neuropsychological data was replicated after excluding patients with major depression (n=2, ADL+; n=4, ADL-) or undefined status of depression (n=1, ADL-). Except for the Go-Nogo TAP task assessing attention (*p*=0.06, see Table S1 for details) differences in the PANDA (*p*=0.01) and in tasks measuring visuo-construction (*p*<0.05) reach statistical significance. 

## Discussion

According to the MDS-TF criteria [3,4], significant impairment of ADL function is the core criterion for differentiating PDND from PDD. We investigated which clinical (including cognitive) factors contribute to the caregiver’s rating of significant ADL dysfunction in PD patients. Following the recommended Level I criteria of the MDS-TF, we selected a patients group with cognitive impairment indicative of PDD [4]. In our sample, 22.9% of PD patients met the cognitive requirements for diagnosis of PDD according to the consensus guidelines, resembling previous data [38]. The selection of the Level-I-cogn group was primarily based on the MMSE and the verbal fluency tasks. Neuropsychological test data verified that PD patients who fulfilled the Level-I-cogn criteria were indeed cognitively more severely impaired than PD patients who did not meet these criteria. This further supports the usefulness of the proposed criteria. Moreover, in line with previous studies [39–41], cognitive dysfunction was associated with older age, longer disease duration and behavioral abnormalities. 

The main focus of this study was to compare the cognitive, neurobehavioral and motor characteristics of PD patients with cognitive impairment according to the Level-I-cogn criteria that were either judged by their caregivers as having (ADL-) or not having (ADL+) ADL impairment. ADL+ and ADL- patients differed in various aspects, namely in neuropsychological performance, motor type, communication problems, and medication. In our study, caregiver ratings closely correlated with self-estimates of PD patients and those of physician. This argues for the validity of the observation and judgment of proxies. Therefore, we suggest that standardized caregiver ratings as used in this study can contribute to the clinical PDD diagnosis. In fact, two different cognitive phenotypes could be differentiated within our patient group with substantial cognitive impairment. 

Performances in attention and visuo-construction tasks were strongly related to the caregivers’ ADL ratings and therefore more pronounced in the ADL- group. These results fit well with previous work. Reduced attention in PD patients is one of the strongest predictors for the severity of caregiver rated ADL impairment [15]. Moreover, impaired visuo-constructional abilities can predict future onset of PDD [2,5]. Yet, our data do not support previous findings of a close relationship between executive function and ADL function in PD [42,43]. This discrepancy might partly be explained by different assessments and recruitment strategies. In contrast to Cahn and colleagues [42] who explicitly focused on the assessment of executive function in candidates for pallidotomy, we examined a broad range of cognitive domains known to be affected in PD in a sample with a pre-defined cognitive status. Dymek and co-workers identified executive dysfunction as the main predictor for loss of capacity to consent to treatment in cognitively impaired PD patients [44]. In this study, however, we revealed attention as the main contributor to caregiver`s judgment on ADL dysfunction. Unlike the work of Dymek and co-workers, we used subjective proxy statements to define ADL dysfunction in PD. 

With regard to non-cognitive symptoms, our results are consistent with previous observations that a higher ADL impairment is more closely related to the PIGD than to the tremor dominant subtype [45]. It has been demonstrated repeatedly that the risk of PDD is increased in PIGD patients, particularly in later disease stages, compared to the other motor subtypes [31,46,47]. Interestingly, in our study, patients with ADL- were not older and showed only a tendency of longer disease duration compared to those with ADL+. Nevertheless, this study adds to the hypothesis that a common or associated pathological process leads to progressive cognitive decline and development of PIGD motor type [6,46]. 

Moreover, we found that ADL- patients reported more communication problems than ADL+ patients. Speech and voice difficulties are common in PD patients [48] and are associated with motor and non-motor symptoms. A self-perceived negative impact on communication is known to be related to PD duration [49] and ADL dysfunction [50]. Further, it is assumed that cortically modulated, executive (dys)function underlies verb production in PD [51], which itself can alter the ability to communicate. As mentioned before, ADL- and ADL+ patients did not differ according to their executive function, but rather in attention. Based on our data we can only speculate that there might also be a link between attention and communication problems or the speech process. 

Almost two thirds of our patients with ADL- were treated with both levodopa and dopaminergic agonists, which revealed a tendency to be taken more often than in ADL+ patients. However interpretation of this effect is limited by the sample size of our cohort. One might speculate that the association of the PIGD subtype with ADL impairment in our cohort explains this effect. 

This is also interesting in the light of existing evidence that PDD patients are more prone to hallucinations and psychosis than PD patients without dementia [52]. Moreover, if these symptoms occur, they are primarily treated with antipsychotics. This might explain our finding that ADL- patients receive such drugs more often although the difference of hallucination frequency between the groups was not statistically significant. 

### Limitations

The present study applied the MDS-TF Level-I screening procedure to define cognitive impairment indicative of PDD [4]. Specificity of this approach is high, however sensitivity may be lower [12,38,41,53]. For the sake of high diagnostic specificity, it could therefore be the case that we did not include all PD patients showing a substantial cognitive impairment. We cannot exclude the fact that caregivers might not be able to detect slight progressive changes in patients ADL function at home, especially if they are masked by accompanying motor or other non-cognitive symptoms (e.g. autonomic failure or psychiatric abnormalities) [8,54]. It is also possible that these conflicting factors modulated the standardized caregiver ADL ratings. 

Moreover, our conclusions based on a group of 30 patients who fulfilled the Level-I-cogn criteria are limited by the sample size and the exploratory nature of the study. Further studies with larger sample sizes are needed to verify our results. 

In contrast to the recommendation of the MDS-TF, patients with major depression were not excluded. We argue that depression affects about 35 percent of PD patients and can influence both, cognitive performance and ADL function [55,56]. 

Therefore, in patients with confirmed major depression, a PDD diagnosis is a possibility [3,4] as discussed in recent validation studies [12,38]. During their daily routine, clinicians are often faced with this co-morbidity, particularly because the frequency of both conditions increase with disease duration [1,57]. Moreover, patients with depression were included in this study, as it presents a possible risk marker for PDD. The occurrence and frequency of depression has been shown to be associated to faster cognitive decline [58]. In our sample the ADL+ and ADL- group did not statistically differ in the BDI total score. Thus, we conclude that that the severity of depression does not have an major effect on caregiver–rated ADL (dys)function as assessed in this study.

## Conclusions

Caregiver ADL ratings corresponded well with the self-impression of PD patients and ratings of physician. This supports the validity of the observation and judgment of proxies. Moreover, caregiver ADL ratings were found to be associated with attention, but also with other symptoms such as PIGD subtype, communication problems and medication in PD patients with cognitive decline indicative of dementia. Our data suggests that not only cognitive factors, but also non-cognitive factors seem to be linked to the diagnosis of PDD associated with ADL-.

## Supporting Information

Table S1 **Neuropsychological assessment PD patients with cognitive impairment indicative for Parkinson’s disease dementia (PDD) who were rated by their caregivers as having an ADL impairment (ADL-** **) or not (ADL+) after the exclusion of patients with major depression (n=2 ADL+, n=5 ADL-)**.
Table S1 shows the replication of the main analysis after excluding patients with major depression (n=2, ADL+; n=4, ADL-) or undefined status of depression (n=1, ADL-). Values are given as median and range (minimum-maximum). Mean group performances are given in relation to the standardized values provided by the test manuals with lower values indicating poorer test performances. Only for the Berlin Apraxia Test raw data are presented. ADL: Activities of Daily Living.; PANDA: Parkinson Neuropsychometric Dementia Assessment; PD: Parkinson's Disease; PDD: Parkinson's Disease Dementia; p: Level of significance; N: number.
(DOCX)
