# Supplementary material for: Clinical Characteristics with an Impact on ADL Functions of PD Patients with Cognitive Impairment Indicative of Dementia
Source: PLoS One. 2013 Dec 9;8(12):e82902. doi: 10.1371/journal.pone.0082902 (PMC3857297; doi:10.1371/journal.pone.0082902)
Supplement: Table S1 — Neuropsychological assessment PD patients with cognitive impairment indicative for Parkinson’s disease dementia (PDD) who were rated by their caregivers as having an ADL impairment (ADL- ) or not (ADL+) after the exclusion of patients with major depression (n=2 ADL+, n=5 ADL-). Table S1 shows the replication of the main analysis after excluding patients with major depression (n=2, ADL+; n=4, ADL-) or undefined status of depression (n=1, ADL-). Values are given as median and range (minimum-maximum). Mean group performances are given in relation to the standardized values provided by the test manuals with lower values indicating poorer test performances. Only for the Berlin Apraxia Test raw data are presented. ADL: Activities of Daily Living.; PANDA: Parkinson Neuropsychometric Dementia Assessment; PD: Parkinson's Disease; PDD: Parkinson's Disease Dementia; p: Level of significance; N: number. (DOCX) [file pone.0082902.s001.docx]

Table S1: Neuropsychological assessment of PD patients with cognitive impairment indicative for Parkinson’s disease dementia (PDD) who were rated by their caregivers as having an ADL impairment (ADL-) or not (ADL+) after the exclusion of patients with major depression (n=2 ADL+, n=5 ADL-)

|  |  |  | **No ADL impairment (ADL+)** |  | **ADL**  **impairment (ADL-)** | **ADL+**  **vs.**  **ADL-** |
| --- | --- | --- | --- | --- | --- | --- |
|  |  | **n** | **values** | **n** | **values** | **p** |
| *Minimental State Examination* | screening | 10 | 24.5 (19-25) | 13 | 24 (15-25) | 0.74 |
| *PANDA* | screening | 10 | 16.50 (9-26) | 13 | 9.00 (7-17) | **0.01** |
| *Tower of London* | problem solving | 10 | 10 (0-35) | 13 | 8 (0-51) | 0.25 |
| *Consortium to Establish a Registry for Alzheimer's Disease* |  |  |  |  |  |  |
| Verbal fluency | word generation | 10 | 4.5 (1-76) | 13 | 16 (0-90) | 0.20 |
| Boston Naming Test | naming | 10 | 5 (0-76) | 13 | 8 (0-95) | 0.55 |
| Word-list memory | memory | 10 | 5.5 (0-42) | 13 | 3 (0-86) | 0.98 |
| Word-list recall | memory | 10 | 21 (1-86) | 13 | 13 (0-69) | 0.59 |
| Word-list recognition | memory | 10 | 3.5 (0-21) | 13 | 12 (0-86) | 0.20 |
| Word-list intrusion | memory | 10 | 3.0 (0-79) | 13 | 3 (0-82) | 0.92 |
| Praxis | visuo-construction | 10 | 4 (0-86) | 13 | 0 (0-24) | **0.043** |
| Praxis-Delay | visuo-construction/memory | 10 | 16.5 (0-84) | 13 | 0 (0-12) | **0.006** |
| Trail Making Test, Part A | psychomotor speed | 10 | 5 (0-62) | 13 | 2.0 (0-38) | 0.38 |
| Trail Making Test, Part B | set shifting | 10 | 0 (0-58) | 13 | 0 (0-66) | 0.84 |
| *Wechsler Memory Scale Revised* |  |  |  |  |  |  |
| Logical Memory I | Memory | 10 | 2.5 (1-12) | 13 | 3 (1-70) | 0.48 |
| Logical Memory II | Memory | 10 | 2.5 (0-47) | 13 | 5.0 (0-68) | 0.79 |
| *Nuernberger-Alters-Inventory* |  |  |  |  |  |  |
| Digit span | Memory | 10 | 27 (4-56) | 13 | 56 (4-100) | 0.15 |
| Figure Test | visuo-spatial/memory | 10 | 20.5 (0-82) | 13 | 20 (0-88) | 0.57 |
| *Visual Object and Space Perception Battery* |  |  |  |  |  |  |
| Object decision | visuo-spatial | 10 | 42.9 (3.6-100) | 13 | 7.1 (2.4-47.6) | 0.07 |
| *Berlin Apraxia Test* | praxis/executive function | 10 | 30.5 (21-38) | 13 | 33.0 (20-39) | 0.27 |
| *Test for Attentional Performance* |  |  |  |  |  |  |
| Alertness-no cue, Median | attention/alertness | 10 | 5.5 (0-46) | 13 | 1.5 (0-50) | 0.07 |
| Alertness-with cue, Median | attention/alertness | 10 | 5.5 (0-50) | 13 | 2.0 (0-31) | 0.23 |
| Go-Nogo, Median | directed attention | 10 | 15.5 (0-66) | 13 | 2.0 (0-54) | 0.06 |
